# Liraglutide and renal mitochondrial homeostasis: targeting the “metabolism-inflammation-lithogenesis” axis in metabolic syndrome

**DOI:** 10.3389/fphar.2026.1903973

**Published:** 2026-09-04

**Authors:** Fei Wang, Qiuyu Li, Quan Wen, Boyan Su, Haoji Zhou, Yuqiang Fu, Hui Chen, Qiqi He

**Affiliations:** 1 Department of Child Healthcare and Rehabilitation, The Second Hospital & Clinical Medical School, Lanzhou University, Lanzhou, China; 2 Department of Endocrinology, The Second Hospital & Clinical Medical School, Lanzhou University, Lanzhou, China; 3 Department of Urology, Key Laboratory of Disease of Urological Systems, Gansu Nepho-Urological Clinical Center, The Second Hospital & Clinical Medical School, Lanzhou University, Lanzhou, China

**Keywords:** liraglutide, metabolic syndrome, mitochondrial dysfunction, nephrolithiasis, NLRP3 inflammasome, oxidative stress, PGC-1α, renal tubular injury

## Abstract

Nephrolithiasis associated with metabolic syndrome represents a growing global public health burden with a 5-year recurrence rate of up to 50%. Current first-line preventive strategies, primarily potassium citrate and thiazide diuretics, only correct urinary chemical abnormalities symptomatically, and patient adherence remains below 50% at 1 year. Importantly, these approaches fail to address the core pathological basis of tubular injury driven by metabolic dysregulation. Mitochondrial dysfunction is the central mechanistic hub linking systemic metabolic stress to intrarenal lithogenic susceptibility. In the setting of metabolic syndrome, impaired mitochondrial biogenesis and excessive mitochondrial reactive oxygen species (mtROS) production in renal tubular epithelial cells activate the NOD-like receptor family pyrin domain containing 3 (NLRP3) inflammasome, establishing a self-perpetuating vicious cycle of “metabolic disturbance, mitochondrial damage, inflammatory amplification, calcium oxalate crystal deposition'. This core pathogenic loop has not been targeted by existing therapeutic strategies. Liraglutide, a long-acting glucagon-like peptide-1 receptor agonist (GLP-1RA), exerts pleiotropic renoprotective effects independent of its canonical glucose-lowering actions. It coordinately activates peroxisome proliferator-activated receptor gamma coactivator-1α (PGC-1α) through two complementary pathways: transcriptional upregulation through the PKA/CREB axis, and post-translational deacetylation via the AMPK/SIRT1 pathway. This dual activation restores mitochondrial homeostasis, reprograms tubular lipid metabolism, attenuates oxidative stress, and suppresses inflammatory cascades. This review is the first to systematically integrate the mitochondrial pharmacology of liraglutide with the pathophysiology of nephrolithiasis. It critically appraises the strengths and limitations of preclinical and clinical evidence, identifies key knowledge gaps in the field, and proposes a phased translational research roadmap encompassing mechanistic validation, biomarker development, and clinical trial design. This work provides a solid theoretical foundation for repurposing GLP-1RAs for the prevention of this condition.

## 1 Introduction

Liraglutide is a long-acting glucagon-like peptide-1 (GLP-1) analogue derived from native human GLP-1 through two structural modifications: a lysine-to-arginine substitution at position 34, and attachment of a 16-carbon palmitoyl fatty acid side chain to the lysine at position 26 via a γ-glutamyl linker. These modifications yield a plasma half-life of approximately 13 h, adequate for once-daily subcutaneous administration. They promote reversible albumin binding and reduce susceptibility to dipeptidyl peptidase-4 (DPP-4) degradation, and maintain high-affinity binding to the GLP-1 receptor (GLP-1R) (Knudsen and Lau, 2019).

Beyond its well-established roles in pancreatic β-cells and the central nervous system, GLP-1R is reported to be expressed across multiple metabolically active tissues, including adipose tissue, liver, skeletal muscle, and the kidney (Szekeres et al., 2024; Iwai et al., 2023). However, it is important to note that the exact intrarenal localization of GLP-1R remains a subject of ongoing debate. Transcriptomic and immunohistochemical studies yield conflicting results regarding GLP-1R distribution between renal tubular epithelial cells and vascular-glomerular compartments (Hinrichs et al., 2024). This broad tissue distribution underpins the multi-organ pharmacodynamic profile of liraglutide, whose actions extend far beyond glycemic control. Via GLP-1R signaling in these tissues, liraglutide modulates appetite, lipid handling and energy expenditure, and exerts direct organ-protective effects. Together, these properties position GLP-1R as a convergent node through which a single agent can simultaneously target metabolic dysregulation, mitochondrial dysfunction, and inflammatory signaling at multiple tissue sites (Rodriguez et al., 2025).

The link between metabolic syndrome (MetS) and nephrolithiasis is now firmly established. Prospective data from Chinese cohorts demonstrate that MetS substantially increases the risk of incident stones, with obesity and hypertension each acting as independent lithogenic factors (Sun X. et al., 2025). Cross-sectional studies from other populations have replicated this pattern, and further work suggests that the metabolic components of MetS may also shift stone composition (Moftakhar et al., 2022; Bu et al., 2025). These observations have reshaped how nephrolithiasis is understood: in patients with MetS, kidney stones are less a primary urological disorder than a local renal manifestation of systemic metabolic dysregulation (Carvalho et al., 2025).

What connects systemic metabolism to intrarenal crystal formation remains an open question, but a growing body of work points to mitochondrial dysfunction as the key interface (Chaiyarit and Thongboonkerd, 2012; Zhuang et al., 2015; Wang et al., 2021; Hou et al., 2024). Within the kidney, impaired mitochondrial biogenesis and fatty acid oxidation in adipose tissue, liver, and skeletal muscle drive the insulin resistance and lipotoxicity that define MetS (Wang et al., 2021; Hou et al., 2024). On the renal side, calcium oxalate crystals damage tubular epithelial mitochondria, inducing oxidative stress and activating the NOD-like receptor family pyrin domain containing 3(NLRP3) inflammasome in a self-amplifying loop that promotes further crystal adhesion and retention (Chaiyarit and Thongboonkerd, 2012; Zhuang et al., 2015). In this sense, mitochondrial dysfunction sits at the crossroads: it is simultaneously a consequence of the metabolic milieu of MetS and a facilitator of the intrarenal lithogenic process (Thompson et al., 2025).

Despite this convergence, the literature on mitochondrial pharmacology in nephrolithiasis remains fragmented. Liraglutide’s effects on mitochondrial homeostasis have been documented in adipose tissue, myocardium, and proximal tubule models (Pedrosa et al., 2022; Rossi et al., 2025; Zhou et al., 2025; Zhao et al., 2015), but these findings have not been integrated through the lens of stone pathophysiology. Whether pharmacological restoration of mitochondrial function through GLP-1R activation can interrupt the cascade from metabolic stress to crystal deposition has not been systematically evaluated. This review addresses that gap. Among the GLP-1 receptor agonist class, liraglutide has the most extensively validated preclinical evidence for mitochondrial biogenesis mediated by GLP-1R and organ protection dependent on PGC-1α across multiple metabolically relevant tissues, including the kidney (Elkhoely, 2023; Wang et al., 2018), adipose tissue (Zhou et al., 2019) and myocardium (Zhang et al., 2021). We therefore selected it as the prototypical agent for this mechanistic review. Using liraglutide as our reference model, we synthesize evidence linking its PGC-1α-dependent pathways to each node of the “mitochondrial metabolism–inflammation–lithogenesis” axis, while noting that the proposed anti-lithogenic mechanisms are likely generalizable to the broader GLP-1RA pharmacological class, given consistent evidence for class-wide renoprotective effects.

Despite the rising prevalence of nephrolithiasis associated with MetS, current preventive strategies remain significantly limited. First-line potassium citrate corrects hypocitraturia, but dosing three to four times daily and prominent gastrointestinal adverse effects result in an adherence rate below 50% at 1 year (Cheungpasitporn et al., 2016), and the agent carries a risk of hyperkalemia in patients with concurrent chronic kidney disease (Papatsoris et al., 2025). Thiazide diuretics exert their effects by reducing urinary calcium excretion, but long-term use can induce electrolyte disturbances, glucose and lipid metabolism abnormalities, and bone loss, further exacerbating the underlying comorbidities in patients with MetS (Carvalho et al., 2025). More importantly, both agents are symptomatic treatments targeting urinary chemical abnormalities and fail to address the core pathological mechanisms of mitochondrial dysfunction and chronic inflammation, which limits their stone-preventive efficacy in this patient population (Thompson et al., 2025).

Against this background, this review first systematically outlines the pathophysiological basis of nephrolithiasis associated with MetS, identifying mitochondrial dysfunction as the central hub linking systemic metabolic dysregulation to intrarenal lithogenesis. It then dissects the molecular pharmacological mechanisms by which liraglutide synergistically activates PGC-1α via the PKA/CREB and AMPK/SIRT1 signaling axes, and its downstream regulatory effects on lipid metabolism, redox balance, and inflammatory responses. Building on this, it integrates liraglutide’s anti-lithogenic effects at three levels: renal tubular epithelial protection, inflammasome inhibition, and urinary chemistry modulation. Subsequently, it objectively evaluates the strength and contradictions of existing clinical evidence and identifies key gaps in current research. Finally, it proposes a complete translational research roadmap from mechanistic validation to clinical application, providing a theoretical basis and practical guidance for repurposing of GLP-1RAs to prevent nephrolithiasis associated with MetS.

## 2 Pathological basis: mitochondrial dysfunction and inflammation in nephrolithiasis associated with metabolic syndrome

### 2.1 Obesity and insulin resistance: core features of MetS and key drivers of stone risk

Central obesity, driven by visceral fat accumulation, is the pathophysiological anchor of MetS (Bushita et al., 2025). Excess saturated fatty acids released from expanded visceral adipose tissue trigger lipotoxic stress in peripheral organs. They activate JNK and nuclear factor-kappa B (NF-κB) pathways through lipid intermediates such as diacylglycerol and ceramide, and attenuate insulin signaling via the IRS-1/PI3K/Akt axis (Lee and Lam, 2019). Over time, this metabolic insult provokes inflammatory infiltration of adipose tissue itself, with sustained release of pro-inflammatory cytokines that promote ectopic lipid deposition in the liver and skeletal muscle. The result is a self-reinforcing cycle in which obesity, inflammation, and insulin resistance (IR) amplify one another (Lee and Lam, 2019; Kobayashi et al., 2020). Notably, this vicious cycle is not confined to older adults; visceral fat accumulation correlates with core MetS components, including dyslipidemia, hypertension and hyperglycemia, even in young populations, underlining its value as an early intervention target (Kobayashi et al., 2020).

For nephrolithiasis, obesity and IR are not merely associations but established, quantifiable risk factors. A pooled analysis of cohort studies has shown that each 5 kg/m^2^ increment in BMI raises the risk of incident stones by 21% (RR 1.21, 95% CI 1.12–1.30), and each 10 cm increase in waist circumference adds a further 16% (RR 1.16, 95% CI 1.12–1.19) (Kobayashi et al., 2020). The mechanistic link runs largely through IR: impaired insulin signaling reduces renal ammonium production and excretion while enhancing citrate reabsorption in the proximal tubule, producing the aciduria and hypocitraturia that together create a urinary milieu permissive to calcium oxalate crystallization (Ho et al., 2022). Dietary patterns common in obesity, including high sodium, high caloric density and high animal protein, further compound this lithogenic environment, as does the elevated risk of urinary tract infection (Carbone et al., 2018). Recent work using surrogate indices of IR, including METS-IR and TyG-BMI, has confirmed a dose-response relationship with stone risk, with the signal most pronounced in men and in patients with overt diabetes (Shen et al., 2024).

Although these data clearly tie obesity and insulin resistance to stone risk, the relationship is more than associative. The urinary chemical abnormalities discussed above, low pH and low citrate, explain why the urine of an insulin-resistant individual is lithogenic, but they do not fully account for why some patients within this metabolically stressed group go on to form stones while others do not. Answering that question requires a closer look at the renal tubular epithelium itself and at the mitochondria that sustain its function.

### 2.2 Mitochondrial dysfunction: the hub connecting systemic metabolic stress to renal lithogenic susceptibility

Mitochondrial dysfunction has been increasingly implicated as a link between systemic metabolic stress and intrarenal lithogenesis (Chaiyarit and Thongboonkerd, 2012; Zhuang et al., 2015; Wang et al., 2021; Hou et al., 2024; Thompson et al., 2025), though whether it plays a causal role and can be therapeutically targeted remains to be determined.

Mitochondria are the primary source of cellular ATP and central regulators of fatty acid oxidation, redox balance, and apoptotic signaling. These functions are indispensable in tissues with high metabolic demand, including adipocytes, skeletal muscle, hepatocytes, and renal tubular epithelial cells (Nunnari and Suomalainen, 2012). In MetS, mitochondrial dysfunction is not confined to a single organ. In adipose tissue, obesity reduces mitochondrial DNA copy number, disrupts cristae architecture, and suppresses the expression of oxidative phosphorylation subunits, locking adipocytes into a state of excess lipid storage and dysregulated lipolysis that persists even after weight reduction (Gonzalez-Franquesa et al., 2022). In skeletal muscle, high-fat feeding and obesity downregulate PGC-1α, limiting mitochondrial biogenesis and creating a bidirectional relationship with insulin resistance: impaired mitochondrial respiration promotes intramyocellular lipid accumulation, while insulin resistance itself further depresses mitochondrial function through attenuated PGC-1α signaling (Cade, 2018; Lee et al., 2017; Goodpaster, 2013; Holloszy, 2013). In the liver, the parallel loss of mitochondrial fatty acid oxidation capacity drives hepatic steatosis and accelerates hepatic insulin resistance; restoring β-oxidation via activation of mitochondrial ACSL1 is sufficient to reverse these abnormalities in experimental models (Zheng. et al., 2025). A common thread runs through all three tissues: mitochondrial compromise is both cause and consequence of the metabolic disarray that defines MetS.

The clinical relevance of this systemic mitochondrial disturbance to nephrolithiasis lies in the kidney’s heavy reliance on oxidative metabolism, particularly in the proximal tubule. Renal tubular epithelial cells (RTECs) are among the cells with the highest mitochondrial content in the body. The proximal tubule, which reabsorbs the bulk of the glomerular filtrate, derives over 90% of its ATP from oxidative phosphorylation (Bhargava and Schnellmann, 2017). This extraordinary metabolic demand renders RTECs acutely vulnerable to mitochondrial injury. In the MetS milieu, systemic lipotoxicity and insulin resistance converge on the proximal tubule, suppressing PGC-1α expression and eroding the mitochondrial reserve that RTECs need to cope with subsequent insults (Bhargava and Schnellmann, 2017). The metabolic environment of MetS lowers the threshold for tubular damage induced by crystals before a single crystal adheres.

When calcium oxalate crystals do encounter the tubular epithelium, they inflict direct mitochondrial damage. Crystal adhesion to RTECs triggers a collapse of mitochondrial membrane potential, a burst of mitochondrial reactive oxygen species (mtROS), and opening of the mitochondrial permeability transition pore, with cyclophilin D activation further amplifying the damage (Yasui et al., 2016; Xu et al., 2024). Damaged mitochondria then release oxidized mitochondrial DNA, which engages the NLRP3 inflammasome and drives inflammation and pyroptosis mediated by IL-1β and IL-18 (Su et al., 2024). The result is a self-perpetuating pathological cycle: metabolic stress and mitochondrial dysfunction drive crystal deposition, which in turn amplifies inflammation and further mitochondrial injury. This cycle not only links systemic metabolism to local lithogenesis but also positions mitochondrial homeostasis as a node where pharmacological intervention could, in principle, interrupt both limbs of the process. That the ROS scavenger Mito-Tempo, which targets mitochondria, significantly reduces crystal deposition and renal injury in experimental models lends experimental credence to this strategy (Xu et al., 2024). It is at this node that liraglutide’s pharmacology becomes directly relevant, as the sections that follow will show.

## 3 Molecular pharmacology: liraglutide and renal mitochondrial homeostasis

**FIGURE 1:**
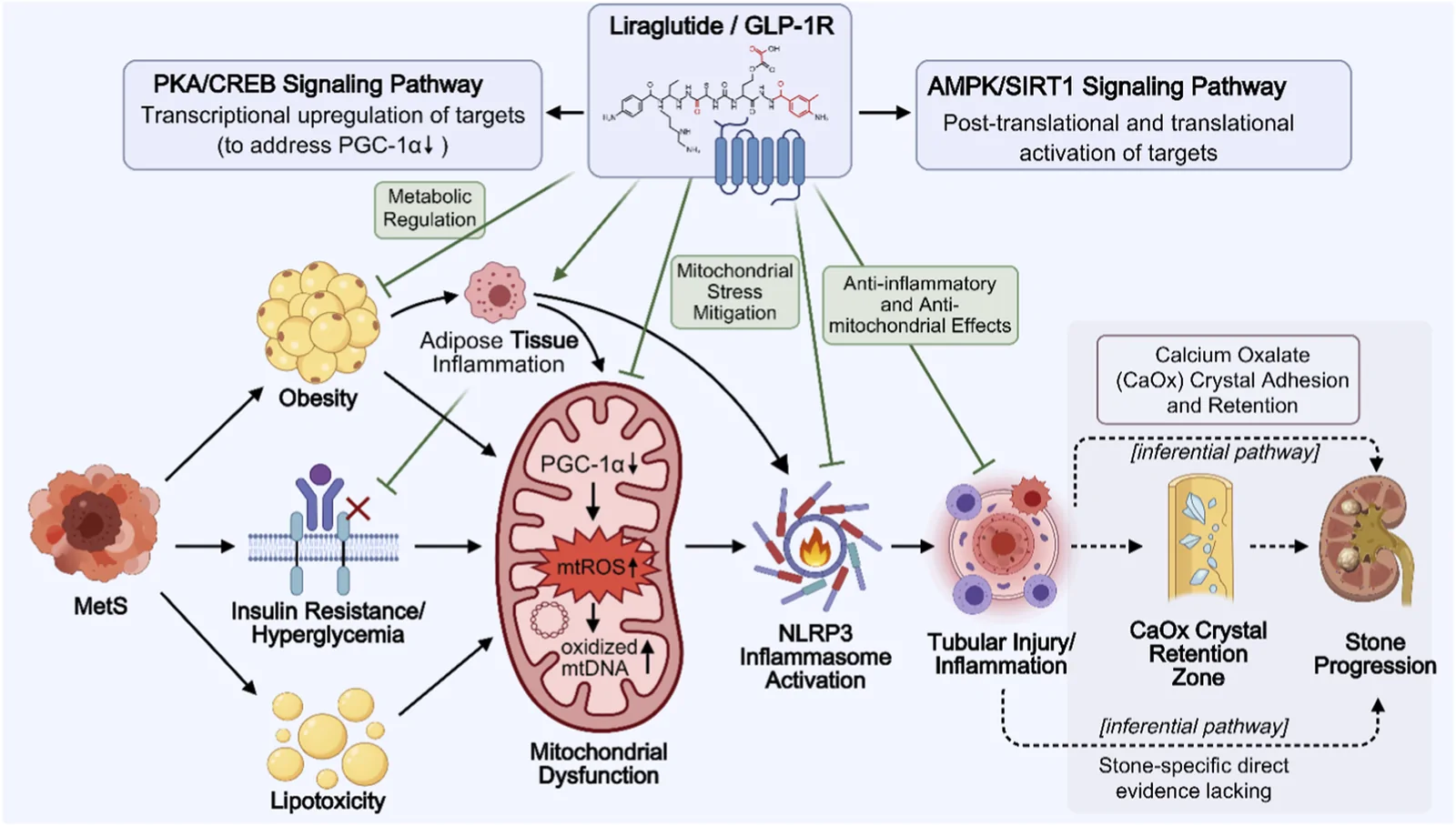
Mechanistic framework of liraglutide for the prevention of nephrolithiasis associated with metabolic syndrome. This schematic illustrates how mitochondrial dysfunction links systemic metabolic abnormalities to intrarenal lithogenesis. MetS drives renal tubular mitochondrial dysfunction via three interconnected pathways: adipose tissue inflammation induced by obesity, insulin resistance/hyperglycemia, and lipotoxicity. Impaired mitochondrial function leads to excessive mtROS production and oxidized mtDNA release, which activates the NLRP3 inflammasome and triggers tubular injury and inflammation, subsequently promoting CaOx crystal adhesion, retention, and eventual stone progression. Liraglutide exerts multi-level protective effects via GLP-1R activation: it upregulates PGC-1α through both transcriptional upregulation mediated by PKA/CREB and post-translational activation mediated by AMPK/SIRT1, thereby restoring mitochondrial homeostasis; it also ameliorates systemic metabolic abnormalities and adipose tissue inflammation, and exerts direct anti-inflammatory and protective effects on mitochondria. Legend: Solid black arrows indicate pathways supported by robust experimental evidence. Dashed black arrows marked with “ [inferential pathway]” represent mechanistically plausible but speculative links lacking direct experimental validation specific to stone disease. Solid green arrows indicate the regulatory and protective effects of liraglutide. Abbreviations: MetS, metabolic syndrome; GLP-1R, glucagon-like peptide-1 receptor; PKA, protein kinase A; CREB, cAMP response element-binding protein; AMPK, AMP-activated protein kinase; SIRT1, sirtuin 1; PGC-1α, peroxisome proliferator-activated receptor gamma coactivator-1α; mtROS, mitochondrial reactive oxygen species; mtDNA, mitochondrial DNA; NLRP3, NOD-like receptor family pyrin domain containing 3; CaOx, calcium oxalate.

Figure 1 depicts the mechanistic framework by which liraglutide interrupts the “mitochondrial metabolism–inflammation–lithogenesis” axis in nephrolithiasis associated with MetS. To clearly differentiate experimentally validated signaling from inferential links specific to stone disease, pathways supported by established evidence are shown as solid arrows, whereas hypothetical components lacking direct validation specific to stone models are indicated by dashed arrows. To connect the pathophysiology of tubular mitochondrial injury (Section 2) with liraglutide’s pharmacology, the drug targets three interdependent defects. It reverses the loss of mitochondrial reserve induced by metabolic stress by driving biogenesis via the PKA/CREB pathway; suppresses mtROS overproduction evoked by crystals overproduction through redox rebalancing mediated by PGC-1α; and inhibits the release of oxidized mtDNA and subsequent NLRP3 inflammasome activation, the principal inflammatory amplifier that promotes crystal retention, via quality control mediated by AMPK/SIRT1. The following subsections dissect the signaling pathways that underpin each of these actions.

### 3.1 PKA/CREB-PGC-1α signaling axis: core pharmacological coupling from receptor activation to mitochondrial biogenesis

Available evidence from various metabolic and renal injury models suggests that liraglutide regulates mitochondrial function primarily through the canonical Gs-cAMP-PKA cascade, which is likely to couple GLP-1R activation to the transcriptional activation of PGC-1α (Elkhoely, 2023; Bao et al., 2015). Upon GLP-1R activation, the resulting increase in intracellular cAMP activates PKA, which subsequently phosphorylates CREB (Bullock and Habener, 1998). Phosphorylated CREB then directly initiates PGC-1α transcription (Hong et al., 2011), and this PKA/CREB/PGC-1α axis can be engaged by liraglutide in renal tissue (Elkhoely, 2023). PGC-1α serves as a central transcriptional coactivator that drives mitochondrial biogenesis and maintains oxidative phosphorylation capacity. In the specific context of nephrolithiasis, preserving this PGC-1α-driven mitochondrial reserve is hypothesized to be critical for renal tubular epithelial cells to withstand the severe metabolic and oxidative stress induced by calcium oxalate crystal adhesion (Zhou et al., 2019; Wu et al., 2022).

The GLP-1R/PKA/CREB/PGC-1α signaling axis represents a highly conserved mechanism for mitochondrial quality control across diverse tissues, translating upstream receptor activation into tissue-specific protective outcomes (Elkhoely, 2023; Zhou et al., 2019; Zhang et al., 2021; Wu et al., 2022).

In the kidney, the first direct *in vivo* validation of this pathway comes from an acute kidney injury rat model induced by gentamicin: liraglutide, administered at doses approximating human clinical exposure, restored PKA/CREB phosphorylation, thereby driving mitochondrial biogenesis, preserving cristae architecture, and attenuating tubular apoptosis; concurrent inhibition of the Notch/Hes1 pathway further suggests an additional derepression mechanism that complements direct PGC-1α transcriptional activation (Elkhoely, 2023).

The pharmacological specificity of this cascade is strongly supported by findings in primary astrocytes, where blockade of GLP-1R, adenylate cyclase and PKA, three mechanistically distinct interventions, each independently abolished downstream CREB phosphorylation (Bao et al., 2015).

While this core signaling module is consistently engaged across tissues, its functional manifestations diverge sharply: it drives structural mitochondrial biogenesis in the renal tubule, promotes white adipose browning in diet-induced obesity models (Zhou et al., 2019), and suppresses pyroptosis mediated by NLRP3 in diabetic cardiomyopathy (Zhang et al., 2021). Thus, while liraglutide recruits the PGC-1α program as a common response to metabolic and oxidative stress across tissues, the ultimate protective outcome depends on the local cellular context: biogenesis in the renal tubule, adipocyte browning in fat, and inflammasome inhibition in the heart (Elkhoely, 2023; Zhou et al., 2019; Zhang et al., 2021; Wu et al., 2022).

While the mechanisms by which GLP-1RAs regulate renal tubular function via the PKA pathway have received preliminary validation, two key limitations remain. First, cAMP generated downstream of the GLP-1 receptor does not signal exclusively through PKA. The exchange protein directly activated by cAMP (Epac) operates in parallel and has been directly implicated in the regulation of proximal tubular NHE3. Using pharmacological inhibitors and cAMP-specific analogs in LLC-PK_1_ renal proximal tubule cells, Carraro-Lacroix et al. demonstrated that exendin-4 suppresses NHE3 activity through a mechanism requiring both PKA and Epac—H-89 (a PKA inhibitor) partially blocked the effect, U-0126 (a MEK/ERK inhibitor downstream of Epac) partially blocked it, and only the combination of both inhibitors fully prevented NHE3 inhibition, establishing that the two cAMP effectors act cooperatively on this transporter (Austin and Tomas, 2026; Carraro-Lacroix et al., 2009). This dual-pathway mechanism translates into divergent clinical effects depending on the pharmacokinetic profile of the GLP-1RA: prolonged treatment with the long-acting agent liraglutide did not affect urine pH, whereas the short-acting agent lixisenatide significantly increased urine pH and urinary sodium excretion, a pattern consistent with more effective engagement of proximal tubular NHE3 inhibition by short-acting agents (Tonneijck et al., 2018). Of note, mitochondrial biogenesis is predominantly mediated by the PKA arm (Bao et al., 2015); whether Epac contributes to other aspects of liraglutide’s renal pharmacology, including NLRP3 inflammasome suppression, remains untested. Given Epac’s distinct modulation of NHE3 and other ion transporters, selective Epac activation may offer a parallel, anti-lithogenic strategy that acts independently of mitochondria. Rather than targeting intracellular metabolic fitness, an approach targeting Epac would directly modify the luminal chemical environment by altering urinary pH and sodium excretion, thereby reducing crystallization propensity. Whether mitochondrial resilience driven by PKA and urinary chemistry modulation mediated by Epac can be jointly harnessed warrants further investigation. Second, all studies that have validated the PKA/CREB/PGC-1α transcriptional axis in the kidney, heart, and adipose tissue were conducted in models unrelated to stone disease. Whether liraglutide recruits this same transcriptional program in the context of calcium oxalate crystal-induced tubular injury is unknown. Nonetheless, the demonstration that this pathway is active in renal tissue and capable of protecting tubular epithelia from oxidative and inflammatory damage offers a clear mechanistic link to the mitochondrial injury described in Section 2.2, and a strong rationale for extending this work into experimental models specific to stone disease.

Based on these non-stone models, the PKA/CREB signaling cascade is hypothesized to represent a primary molecular link between GLP-1R activation and PGC-1α-mediated mitochondrial biogenesis in the kidney (Elkhoely, 2023; Zhou et al., 2019; Zhang et al., 2021; Wu et al., 2022). In this hypothesized framework, following GLP-1R engagement at the plasma membrane, sequential activation of Gas, adenylyl cyclase, and PKA would lead to the phosphorylation of CREB at Ser133. Phosphorylated CREB in turn transactivates PGC-1α, the transcriptional regulator that initiates the mitochondrial biogenesis program.

Importantly, this pathway exerts its effects primarily at the transcriptional level by increasing PGC-1α mRNA abundance. However, newly synthesized PGC-1α protein is functionally inactive and requires post-translational modification to become fully active and capable of coordinating mitochondrial biogenesis. The AMPK/SIRT1 axis, which governs these key modifications and mitochondrial quality control, is discussed in the following section.

### 3.2 AMPK/SIRT1–PGC-1α axis: post-translational activation

Where the PKA/CREB pathway described in Section 3.1 controls PGC-1α abundance, the AMP-activated protein kinase (AMPK)/sirtuin 1 (SIRT1) axis governs PGC-1α functional activity. Newly synthesized PGC-1α is acetylated at multiple lysine residues and remains transcriptionally repressed until deacetylated by SIRT1, an NAD^+^-dependent deacetylase (Rodgers et al., 2005; Cantó et al., 2009a).

In metabolically active extrarenal tissues, available data suggest that liraglutide activates SIRT1 primarily through AMPK (Zhou et al., 2019; Zhang et al., 2021; Cantó et al., 2009b; Cantó et al., 2010). Activated SIRT1 then deacetylates PGC-1α, converting it into a transcriptionally active conformation that engages the transcriptional machinery and drives downstream gene expression (Rodgers et al., 2005; Cantó et al., 2009a).

The first demonstration that liraglutide engages this signaling cascade was reported in adipose tissue, where Zhou et al. showed that it mediates beige adipogenesis and improves mitochondrial function in diet-induced obese mice (Zhou et al., 2019). Wang et al. subsequently extended this axis to the kidney: in obese rats fed a high-fat diet (HFD), liraglutide increased renal NAD^+^ levels, restored SIRT1/AMPK/PGC-1α pathway signaling, and partially rescued impaired mitochondrial function (Wang et al., 2018). A parallel finding was reported in cardiac tissue, where Zhang et al. showed that liraglutide suppresses pyroptosis dependent on the NLRP3 inflammasome via the SIRT1/AMPK pathway in a PGC-1α-dependent manner (Zhang et al., 2021).

Whether liraglutide activates this same signaling cascade in the context of renal tubular injury induced by calcium oxalate crystals has yet to be investigated. However, convergent findings from multiple tissues and disease models point to its likely involvement. The same AMPK/SIRT1/PGC-1α module is engaged in each tissue where liraglutide confers mitochondrial protection, including adipose tissue, myocardium and kidney, across models of obesity, diabetes, and chronic kidney disease (CKD) (Wang et al., 2018; Zhou et al., 2019; Zhang et al., 2021). Furthermore, this axis has been biochemically validated in proximal tubular cells under metabolic stress (Wang et al., 2018). This offers a strong mechanistic basis for examining this axis in nephrolithiasis associated with MetS, a condition in which tubular ectopic lipid deposition is a core pathological feature.

Thus, these two pathways function as complementary regulators of PGC-1α: one governs transcriptional output (quantity control), and the other governs functional activation dependent on deacetylation (quality control). The full mitochondrial biogenesis program requires that both conditions be met: adequate PGC-1α protein abundance and a sufficient fraction of that protein in the deacetylated, active state. This dual-input regulatory architecture explains why liraglutide, by simultaneously activating both cascades, provides more robust mitochondrial protection than interventions targeting only one arm of the PGC-1α regulatory network.

### 3.3 Downstream consequences: mitochondrial homeostasis and NLRP3 restraint

PGC-1α activation directly counteracts the “metabolism–inflammation–lithogenesis' cycle through three coordinated mechanisms.

#### 3.3.1 Lipid metabolic reprogramming

Activated PGC-1α drives mitochondrial biogenesis and fatty acid β-oxidation, reducing the ectopic tubular lipid deposition characteristic of nephrolithiasis associated with MetS (Wen et al., 2025; Su et al., 2020). Our group previously showed in a HFD-fed rat model that tubular lipid accumulation correlates positively with papillary crystal burden and coexists with mitochondrial structural damage and impaired β-oxidation (Wen et al., 2025). Liraglutide reverses this pattern by promoting lipolysis and inhibiting lipogenesis through the AMPK/SIRT1 axis (Su et al., 2020; Yu P. et al., 2019).

#### 3.3.2 Redox rebalancing

Biogenesis driven by PGC-1α produces mitochondria with efficient respiratory coupling, resulting in less electron leak and lower mtROS output. In parallel, liraglutide activates nuclear factor erythroid 2-related factor 2 (Nrf2), inducing superoxide dismutase, heme oxygenase-1, catalase, and manganese superoxide dismutase (MnSOD), while suppressing NADPH oxidase/NOX4 to limit ROS at the source (Elsiad et al., 2025; Tong et al., 2016; Ying et al., 2023; Zhang et al., 2020; Zhang et al., 2019). We previously confirmed that targeted mtROS scavenging with Mito-Tempo attenuates crystal deposition and tubular injury, validating mtROS suppression as an anti-lithogenic mechanism (Su et al., 2024).

#### 3.3.3 NLRP3 inflammasome suppression

Calcium oxalate crystals trigger mitochondrial damage, releasing a repertoire of damage-associated molecular patterns (DAMPs) that activate the NLRP3 inflammasome via distinct, non-redundant pathways. Oxidized mitochondrial DNA (mtDNA) directly engages the inflammasome complex; extracellular ATP activates the P2X7 receptor to induce K^+^ efflux, a well-established activating signal for NLRP3 (Mariathasan et al., 2006); cardiolipin translocates to the outer mitochondrial membrane and physically recruits NLRP3 (Iyer et al., 2013); and mtROS support both the priming and assembly steps required for full inflammasome activation (Su et al., 2024; Liu et al., 2021; Mulay et al., 2013; Zhou et al., 2011). Through this multi-hit mechanism, interaction between crystals and mitochondria generates multiple parallel activating signals that fuel a self-amplifying inflammatory cascade. Liraglutide interrupts this loop by preserving mitochondrial integrity and reducing mtROS release, as demonstrated in liver and cardiac models (Yu X. et al., 2019; Zhu et al., 2018). Beyond mitochondrial protection, Nrf2 activation and NOX4 suppression further limit the ROS pool required for NLRP3 priming (Elsiad et al., 2025; Tong et al., 2016; Ying et al., 2023).

PGC-1α activation thus counteracts the lithogenic cycle through three coordinated actions: lipid clearance, redox rebalancing, and NLRP3 inhibition. While each of these effects has been demonstrated independently in non-stone models, their combined action has not been tested in systems specific to stone disease. Whether these three mechanisms operate in concert to counteract tubular injury induced by calcium oxalate crystals remains to be experimentally determined. Experimental models of stone disease will be needed to assess whether the signaling framework outlined in this chapter translates to anti-lithogenic efficacy at the tissue level.

## 4 Systemic pharmacology: multi-level intervention in the lithogenic cascade

Evidence from related disease models suggests that liraglutide activates PGC-1α via the PKA/CREB and AMPK/SIRT1 pathways, and that this activation is likely to contribute to improved lipid handling, rebalanced redox status, and restrained NLRP3 activity. Extrapolating to nephrolithiasis, these effects suggest that liraglutide could hypothetically intervene at multiple points along the lithogenic cascade (Elkhoely, 2023; Wang et al., 2018; Zhou et al., 2019; Zhang et al., 2021; Ying et al., 2023; Yu X. et al., 2019; Zhu et al., 2018). This section examines the pharmacological evidence for such an intervention at three levels: tubular epithelial protection, inflammasome suppression, and modulation of urinary chemistry.

### 4.1 Protection of renal tubular epithelial cells

RTEC injury is a prerequisite for crystal adhesion. Liraglutide protects RTECs through mechanisms dependent on GLP-1R. Zhao et al. showed that GLP-1R is expressed in HK-2 cells, that high glucose downregulates it, and that liraglutide reverses this suppression in a dose-dependent manner; co-administration of the antagonist exendin (9–39) abolished the protective effects, confirming receptor dependence (Zhao et al., 2015). While these *in vitro* findings in HK-2 cells and the associated antagonist studies suggest a direct tubular effect, the *in vivo* localization of GLP-1R in the kidney remains controversial (Hinrichs et al., 2024). The lack of consensus largely stems from technical limitations, particularly the insufficient specificity of commercially available anti-GLP-1R antibodies (Mann and Muskiet, 2021). Recent studies using validated approaches indicate that renal GLP-1R expression is predominantly localized to vascular smooth muscle cells rather than the tubular epithelium (Bjørnholm et al., 2021). If tubular GLP-1R expression is indeed negligible *in vivo*, the protective effects of liraglutide described here may be driven primarily by indirect mechanisms, including systemic metabolic improvements, hemodynamic changes, or paracrine signaling from adjacent vascular cells, rather than direct tubular action. In the same study, liraglutide reduced apoptosis induced by high glucose, caspase-3 activation, and tubular structural damage in diabetic rats (Zhao et al., 2015). Zhou et al. further demonstrated that liraglutide downregulates necroptosis mediated by receptor-interacting protein kinase 3 (RIPK3) and mixed lineage kinase domain-like protein (MLKL) via the GLP-1R/PI3K/Akt pathway, limiting metabolically stressed tubular cell death (Zhou et al., 2023). Liraglutide also corrects pathological autophagic imbalance by suppressing the aberrant elevation of LC3-II and Beclin1 observed under high glucose conditions, without blocking basal autophagy (Zhao et al., 2015). On the anti-inflammatory front, liraglutide inhibits activation of NF-κB and mitogen-activated protein kinase (MAPK) mediated by tumor necrosis factor-alpha (TNF-α) (Ye et al., 2019) and downregulates the lectin-like oxidized low-density lipoprotein receptor-1 (LOX-1)/NOX4/NF-κB axis, reducing ROS and inflammatory cytokine release (Ying et al., 2023).

Liraglutide thus engages a receptor-dependent triad of anti-apoptotic, anti-necroptotic and anti-inflammatory actions that preserves tubular integrity under metabolic stress. As RTEC injury gates crystal adhesion, this triad constitutes the first layer of a pharmacologically plausible anti-lithogenic defense.

### 4.2 Restraint of the NLRP3 inflammasome

Beyond preserving tubular epithelial integrity, liraglutide exerts potent anti-inflammatory effects via inhibition of the NLRP3 inflammasome, a critical mediator of renal injury and stone progression induced by crystals (Mulay et al., 2013).

Direct evidence for NLRP3 inhibition mediated by liraglutide in renal tissue comes from multiple preclinical models. In obese rats fed a high-fat diet, liraglutide treatment significantly reduced renal expression of NLRP3, apoptosis-associated speck-like protein containing a CARD (ASC), and cleaved caspase-1, with corresponding decreases in interleukin-1β (IL-1β) and interleukin-18 (IL-18) secretion (Wang et al., 2018). These effects were associated with improved mitochondrial function and reduced tubular injury, and were completely abolished by the GLP-1R antagonist exendin (9–39), confirming receptor dependence. In a rat model of renal ischemia-reperfusion injury, liraglutide attenuated NLRP3 inflammasome activation and tubular pyroptosis via the SIRT1/PGC-1α pathway (Ye et al., 2022). *In vitro* studies in human renal tubular epithelial (HK-2) cells further demonstrated that liraglutide inhibits NLRP3 activation induced by high glucose by enhancing autophagic clearance of damaged mitochondria (Zhao et al., 2015).

While these findings establish that liraglutide suppresses the NLRP3 inflammasome in renal tissue, direct validation in calcium oxalate stone models has yet to be performed. However, the mitochondrial NLRP3 axis driving tubular inflammation induced by crystals is identical to that targeted by liraglutide in other renal disease contexts (Su et al., 2024; Mulay et al., 2013). The ability of liraglutide to preserve mitochondrial integrity and reduce DAMP release is therefore likely to interrupt the inflammatory amplification loop that promotes crystal adhesion and retention.

Confirming this hypothesis and quantifying the relative contribution of inflammasome inhibition to the overall anti-lithogenic effect will require parallel measurement of crystal burden and NLRP3 activity in both renal epithelia exposed to CaOx and *in vivo* stone models. This remains an important experimental priority.

### 4.3 Indirect modulation of the lithogenic urinary environment

Beyond local renal actions, liraglutide may influence stone risk systemically through improved insulin sensitivity and its downstream effects on urinary chemistry.

Insulin resistance impairs renal ammonium excretion and enhances proximal tubular citrate reabsorption, producing the aciduria and hypocitraturia that favor calcium oxalate and uric acid crystallization (Ho et al., 2022; Shen et al., 2024). Ameliorating insulin resistance could, in principle, partially reverse these abnormalities. The clinical evidence, however, is mixed.

Importantly, a retrospective study of 510 stone formers demonstrated that diabetic patients treated with GLP-1RAs had significantly higher 24-h urinary citrate excretion than both diabetic non-users (928 vs. 545 mg; *P* = 0.007) and non-diabetic stone formers (928 vs. 567 mg; *P* < 0.0001). No significant differences in baseline BMI or HbA1c were observed between patients treated with GLP-1RAs and untreated diabetic groups, indicating a stone-preventive effect independent of glycemic control (Zhao et al., 2023). A Medicare claims analysis similarly reported higher urinary citrate among GLP-1RA users versus matched diabetic controls (659 vs. 592 mg, *P* < 0.001) (Hsi et al., 2024). Lixisenatide, a short-acting GLP-1RA, increased urinary pH after 8 weeks of treatment in type 2 diabetes, an effect attributed to NHE3 inhibition in the proximal tubule (Tonneijck et al., 2019); the same group documented accompanying increases in urinary sodium excretion (Tonneijck et al., 2018).

On the other hand, the most direct evidence, a 24-h urine study of 44 obese stone formers losing weight with GLP-based therapies. The study found that while urine oxalate declined from 40 to 32 mg/day (*P* = 0.002), there were no significant changes in urinary calcium, citrate, uric acid, pH, or volume; correspondingly, supersaturation indices for calcium oxalate, calcium phosphate, and uric acid did not change (Feghali et al., 2024). A larger cross-sectional Medicare analysis (349 GLP-1RA users) found no significant difference in any 24-h urine parameter between GLP-1RA users and matched controls after adjustment for multiple comparisons (Schaub et al., 2025).

Several factors likely account for these discrepant findings. First, substantial heterogeneity exists across study populations: studies demonstrating a citrate-raising effect exclusively enrolled patients with diabetes, whereas neutral studies predominantly recruited obese stone formers or used unselected real-world cohorts. Second, pharmacodynamic differences within the GLP-1RA class contribute to variable outcomes: liraglutide, a long-acting GLP-1RA, did not alter urinary pH in a 12-week clinical trial, whereas the short-acting lixisenatide consistently increased urinary pH (Tonneijck et al., 2018; Tonneijck et al., 2019). Finally, most available evidence is derived from retrospective, underpowered studies that were not prospectively designed to evaluate urinary chemistry parameters as primary endpoints.

Current evidence supports a “do no harm' profile for liraglutide: it does not exacerbate lithogenic urinary chemistry, in distinct contrast to malabsorptive bariatric surgery which causes marked hyperoxaluria (Feghali et al., 2024). When combined with its established effects of tubular protection and inflammasome inhibition, this phenotype positions liraglutide as a high-priority candidate for nephrolithiasis associated with MetS. Dedicated prospective trials are required to demonstrate synergistic reduction in clinical stone events.

## 5 From mechanistic hypothesis to translational verification: a roadmap

The preceding sections laid out a pharmacological framework in which liraglutide, via PGC-1α activation dependent on the PKA/CREB and AMPK/SIRT1 pathways, engages multiple nodes of the “metabolism–inflammation–lithogenesis” axis. Whether this framework translates into clinically meaningful stone prevention remains unanswered. This section maps the path from current evidence to future verification: what is known, what is inferential, and what specific experiments could close the gap.

### 5.1 Clinical evidence for GLP-1RAs in nephrolithiasis: strengths and limitations

The most direct evidence on whether GLP-1RAs influence stone risk comes from real-world data and a small number of dedicated 24-h urine studies, rather than randomized trials designed to address this question.

The LEADER trial (n = 9,340) and its CKD subgroup analysis (estimated glomerular filtration rate <60 mL/min/1.73 m^2^, n = 2,158) established the cardiovascular safety and renal tolerability of liraglutide, but nephrolithiasis was not a prespecified endpoint (Davies et al., 2015; Mann et al., 2020). The SCALE Diabetes trial similarly omitted stone events from its standardized adverse event collection (Davies et al., 2015). This absence is not a negative finding: stone outcomes were simply never measured.

Two complementary lines of evidence are available instead. The first comes from large-scale comparative effectiveness studies. Yeh et al. analyzed 482,284 patients with type 2 diabetes in the TriNetX network and found that sodium-glucose cotransporter 2 (SGLT2) inhibitor users had a modestly lower nephrolithiasis risk than GLP-1RA users (HR 0.90; 95% CI 0.86–0.94) (Yeh et al., 2025). A Danish national registry study reported a similar direction (HR 0.51; 95% CI 0.37–0.71) (Kristensen et al., 2021). A 2024 Canadian population-based target trial emulation of 20,146 patients with type 2 diabetes and pre-existing nephrolithiasis found that SGLT2 inhibitor users had a 33% lower rate of recurrent nephrolithiasis compared with GLP-1RA users (105.3 vs. 156.4 events per 1,000 person-years; adjusted rate ratio 0.67, 95% CI 0.57–0.79; number needed to treat = 20) (McCormick et al., 2024). These studies have a critical interpretive boundary: they compare GLP-1RAs against SGLT2 inhibitors, not against placebo or untreated controls. They can tell us that GLP-1RAs do not show a superior stone risk profile compared with SGLT2 inhibitors, but cannot determine whether they reduce, increase, or leave unchanged the absolute stone risk relative to the natural history of the disease.

The second line comes from dedicated 24-h urine studies. Here the evidence is mixed. Schaub et al. analyzed Medicare data from 349 GLP-1RA users with stone disease and found no significant changes in any 24-h urine parameter compared with matched controls; the authors concluded that GLP-1RAs “were not associated with changes that would reduce stone risk” (Zhao et al., 2023; Hsi et al., 2024). Feghali et al. studied 44 obese stone formers losing weight with GLP-1-based therapies and reported a significant decline in urinary oxalate (from 40 to 32 mg/day, *P* = 0.002), but no change in calcium, citrate, uric acid, pH, or volume, and correspondingly no improvement in supersaturation indices for calcium oxalate, calcium phosphate, or uric acid (Feghali et al., 2024). By contrast, a retrospective study of 510 stone formers found diabetic patients receiving GLP-1RAs had higher 24-h urinary citrate than diabetic non-users (928 vs. 545 mg, *P* = 0.007) (Zhao et al., 2023), and a Medicare analysis reported a similar, though smaller, citrate elevation (659 vs. 592 mg, *P* < 0.001) (Hsi et al., 2024).

Ganesan et al. conducted a 2025 propensity score-matched cohort study of 30,954 adults comparing the risk of incident nephrolithiasis between patients initiating semaglutide versus metformin. Over a median follow-up of 2.1 years (780 days), no significant difference in incident stone risk was observed between groups (HR 0.94, 95% CI 0.83–1.06, *P* = 0.30), despite significantly greater weight loss in the semaglutide cohort (Ganesan et al., 2025). Notably, this study included individuals without diabetes, providing the first large-scale controlled evidence that any potential nephrolithiasis-protective effect of GLP-1RAs, if present, is likely modest and cannot be attributed solely to weight reduction.

Several interim conclusions can be drawn from current evidence. Randomized trials of liraglutide have not evaluated nephrolithiasis as a formal endpoint, leaving its stone risk uncharacterized (Davies et al., 2015; Mann et al., 2020). GLP-1RAs do not induce lithogenic urinary changes; stable 24-h urine parameters and increased citrate levels further validate this safety feature (Zhao et al., 2023; Hsi et al., 2024; Feghali et al., 2024; Schaub et al., 2025). Unlike malabsorptive bariatric surgery, which markedly raises urinary oxalate, GLP-1RAs offer a safer option for weight reduction (Feghali et al., 2024). Though favorable urinary profiles suggest a protective effect against stones (Schaub et al., 2025; Yeh et al., 2025), causality remains unproven. These findings provide solid rationale for the mechanistic research and prospective trials described herein. In this context, a recent clinical observation further supports this research direction: patients with nephrolithiasis have markedly reduced peripheral blood mtDNA copy number, which correlates inversely with stone burden (Xu et al., 2024). This evidence reinforces the translational value of targeting mitochondrial dysfunction, including interventions based on GLP-1RAs, for the prevention and management of nephrolithiasis.

### 5.2 Testing the hypothesis: mechanistic validation

This review centers on the hypothesis that liraglutide reduces renal stone susceptibility by restoring tubular mitochondrial homeostasis in a manner dependent on PGC-1α. This hypothesis is amenable to rigorous testing at multiple levels, using well-validated genetic and pharmacological tools.

The gold-standard approach to testing this hypothesis employs a nephron-specific, doxycycline-inducible PGC-1α knockout system. The Pax8-rtTA/tetO-Cre × PGC-1α^^flox/flox^ mouse line, originally termed NiPKO (nephron-specific PGC-1α knockout), confines Cre-mediated recombination exclusively to all major renal tubular segments (proximal tubule, distal tubule, and collecting duct) while sparing extrarenal tissues (Svensson et al., 2016; Traykova-Brauch et al., 2008). Crossing this line with a validated composite model of nephrolithiasis associated with MetS, which combines obesity induced by a high-fat diet with hyperoxaluria induced by ethylene glycol, will directly test whether liraglutide’s anti-lithogenic effect is strictly dependent on tubular PGC-1α. If liraglutide reduces papillary crystal burden in wild-type littermates but not in NiPKO mice, the causal role of PGC-1α is confirmed. This system can also address the converse question: whether tubular-specific transgenic overexpression of PGC-1α is sufficient to phenocopy liraglutide’s protection, establishing sufficiency alongside necessity.

Complementary pharmacological studies can distinguish the distinct contributions of the two upstream signaling cascades that converge on PGC-1α. The selective PKA inhibitor H89 (N-[2-(p-bromocinnamylamino)ethyl]-5-isoquinolinesulfonamide), with a half-maximal inhibitory concentration (IC_50_) of approximately 48 nM for PKA, has been extensively used in renal epithelial cells to block CREB phosphorylation and mitochondrial fission dependent on PKA (Carmel et al., 1990; Puri et al., 2018). This inhibitor enables direct testing of whether PKA/CREB signaling primarily regulates PGC-1α transcription, as predicted by the mechanistic framework in Section 3.1. The highly selective SIRT1 inhibitor EX527 (selisistat), with an IC_50_ of approximately 98 nM, shows high selectivity for SIRT1 over other sirtuin isoforms and is widely used to probe SIRT1-dependent pathways. In renal ischemia-reperfusion models, EX527 abrogates PGC-1α deacetylation mediated by SIRT1 and exacerbates mitochondrial dysfunction, consistent with the SIRT1–PGC-1α axis described in Section 3.2 (Ye et al., 2022). Co-administering these inhibitors with liraglutide in the composite model of MetS and nephrolithiasis will reveal whether one pathway dominates specific functional endpoints: for example, whether PKA/CREB blockade primarily reduces total PGC-1α protein levels, while SIRT1 inhibition preferentially impairs PGC-1α deacetylation and transcriptional activity. Parallel experiments with the antioxidant Mito-Tempo, which is targeted to mitochondria and specifically scavenges mtROS and attenuates renal calcium oxalate crystal deposition and tubular injury *in vivo* (Xu et al., 2024), will further delineate whether the protection conferred by liraglutide is mediated primarily through mitochondrial pathways or involves additional non-mitochondrial signaling dependent on GLP-1R.

A final set of experiments addresses a central unresolved question: whether the tubular protection afforded by liraglutide is direct or merely secondary to weight loss and systemic metabolic improvement. Previous work has demonstrated that kidney-specific GLP-1R deletion exacerbates renal injury in both diabetic and non-diabetic models, establishing that local GLP-1R signaling mediates renoprotection independently of systemic metabolic changes (Zhao et al., 2024). The ongoing controversy over tubular GLP-1R localization makes this targeted validation particularly relevant. Building on these findings, a renal tubule-specific GLP-1R knockout line (Pax8-rtTA/tetO-Cre × GLP-1R^^flox/flox^) can be generated and treated with liraglutide in the same composite model of MetS and nephrolithiasis. This experiment would simultaneously test the hypothesis and provide functional insight into the receptor localization debate. If liraglutide reduces crystal deposition in wild-type littermates but not in tubule-specific GLP-1R knockouts, despite equivalent weight loss and glycemic control in both groups, this would directly isolate the renal component of the anti-lithogenic effect of liraglutide. A complementary approach will address the same question from the opposite direction: administering liraglutide to animals that have already achieved equivalent weight loss via caloric restriction or bariatric surgery, thereby eliminating weight loss as a confounding variable. Such experiments should clarify the relative contribution of direct renal GLP-1R signaling versus indirect systemic metabolic benefits to liraglutide’s potential anti-lithogenic effects.

### 5.3 Toward clinical translation: biomarkers, trial design, and combination strategies

Translating preclinical mechanistic insights into clinical benefit demands a structured, stepwise strategy, tempered by considerable caution. Historically, interventions targeted to mitochondria, including antioxidants specific to mitochondria and biogenesis modulators, have consistently shown robust renoprotective effects in rodent models of acute and chronic kidney injury, yet have repeatedly failed to replicate these benefits in human clinical trials (Patel et al., 2026; Sun A. et al., 2025). Three core factors account for this persistent translational gap in nephrology. First, interspecies differences in metabolic plasticity are substantial: the human proximal tubule relies on distinct compensatory pathways that may blunt the efficacy of single-node interventions (Bhargava and Schnellmann, 2017). Second, achieving sustained, therapeutically active drug concentrations within the mitochondrial compartment of human renal tubular cells remains a major pharmacokinetic barrier. Many preclinical studies use supra-pharmacological doses to elicit robust effects, and whether these mechanisms are engaged at standard clinical dose (for liraglutide, 1.2–3.0 mg/day) remains unproven. Third, the pathophysiological complexity of MetS constrains the impact of isolated mitochondrial targeting; upstream lipotoxic and glycemic insults continue to drive tubular damage unless addressed simultaneously (Sun A. et al., 2025). Recognizing this track record of translational failure underscores the necessity of the rigorous, phased roadmap proposed below, where clinical advancement is strictly gated by objective pharmacodynamic evidence.

The first translational priority is biomarker development. To date, no validated pharmacodynamic biomarker captures renal mitochondrial PGC-1α activity in patients treated with GLP-1RAs. Several candidates merit rigorous prospective investigation. Urinary mtDNA copy number, quantifiable via quantitative PCR in cell-free urine (Eirin et al., 2016), and circulating fibroblast growth factor 21 (FGF21) and growth differentiation factor 15 (GDF15), both established downstream effectors of the GLP-1R/PGC-1α axis, are plausible surrogates. In a prospective cohort of patients with MetS initiating liraglutide, serial measurement of these markers at baseline and 12–24 weeks, paired with 24-h urine chemistry testing, could identify patients with favorable mitochondrial responses who exhibit concurrent improvements in lithogenic urinary parameters.

The second core translational priority is clinical trial design. Stone events should be incorporated as prespecified exploratory endpoints in all ongoing and future cardiovascular outcome trials of GLP-1RAs. Even a *post hoc* analysis of the LEADER and SCALE databases for adverse events related to stones or procedural codes could yield an early signal. A dedicated prospective trial enrolling MetS patients with recurrent calcium oxalate stones, randomized to liraglutide versus standard care, with stone recurrence rate as the primary endpoint and changes in 24-h urine parameters and crystal burden assessed by imaging as secondary endpoints, would provide the most definitive evidence (Papatsoris et al., 2025), although its large required sample size and prolonged follow-up make this a longer-term undertaking.

The third core translational priority is combination strategies. Given the multifactorial pathogenesis of nephrolithiasis associated with MetS, monotherapy is rarely sufficient to target all components of the disease process. Two clinically actionable combinations merit rigorous prospective investigation. First, GLP-1RAs combined with SGLT2 inhibitors, which independently increase urinary volume and citrate excretion in stone formers (Zhao et al., 2023; Hsi et al., 2024) and reduce stone risk in real-world cohorts (Yeh et al., 2025); second, liraglutide combined with potassium citrate, where the former targets upstream metabolic drivers of lithogenesis and the latter directly corrects downstream hypocitraturia (Papatsoris et al., 2025). Whether these combinations produce additive or synergistic anti-lithogenic effects requires dedicated study.

### 5.4 Limitations of this review

Several important limitations should be acknowledged in this narrative review. First, our literature search was limited to peer-reviewed articles published in English and Chinese, introducing a potential risk of language bias. Second, as is common in narrative reviews, we did not register a prospective review protocol or employ standardized risk-of-bias assessment tools, which may have introduced unavoidable subjective judgment during evidence synthesis. Third, key mechanistic inferences, particularly those regarding PKA/CREB and AMPK/SIRT1 signaling in renal tubular epithelium, were extrapolated from studies in non-renal tissues including liver, myocardium and adipose tissue, and have not yet been directly validated in experimental systems specific to stones.

These limitations do not invalidate the proposed mechanistic framework but rather define its current evidential boundaries. To provide a transparent assessment of the strength and limitations of evidence supporting each core claim, we present two summary tables. Table 1 summarizes the overall level of evidence and certainty of conclusions for each major interventional effect of liraglutide. Table 2 presents a detailed stratified analysis of each mechanistic claim, including the source of evidence, specificity to stone-specific experimental models, and key study limitations.

**TABLE 1:** Summary of evidence levels for core interventional effects of liraglutide.

Interventional effect module	Highest level of evidence	Key supporting references	Certainty of conclusion
Weight loss, improvement of IR and dysregulation of glycolipid metabolism	Level 1 (Large-sample multicentre randomized controlled trial, RCT)	Zhou et al. (2019), Davies et al. (2015), Mann et al. (2020), Mashayekhi et al. (2024)	High certainty
Protective effect on RTECs	Level 3 (*In vivo* animal experiment)	Wang et al. (2021), Zhao et al. (2015), Elkhoely (2023)	Moderate certainty
Regulatory effect on renal tubular mitochondrial homeostasis	Level 3 (*In vivo* animal experiment)	Wang et al. (2021), Elkhoely (2023), Wang et al. (2018), Zhou et al. (2019)	Moderate certainty
Inhibitory effect on the NLRP3 inflammasome	Level 3 (*In vivo* animal experiment and *in vitro* cell experiment)	Zhao et al. (2015), Yu X. et al. (2019), Zhu et al. (2018)	Moderate certainty
Interventional effect on calcium oxalate crystal adhesion/deposition	Level 4 (Intervention targeted to mitochondria proven effective; no direct liraglutide data)	Xu et al. (2024)	Very low certainty
Clinical preventive and therapeutic effect on nephrolithiasis incidence/recurrence	Level 4 (Single-centre retrospective observational study)	Yeh et al. (2025), McCormick et al. (2024), Ganesan et al. (2025), Di Scipio et al. (2025)	Very low certainty

**TABLE 2:** Stratified evidence table for claims regarding the role of liraglutide in nephrolithiasis associated with MetS.

Claim of action	Source of evidence	Whether it is specific evidence of stones	Main conclusion	Main limitations
Liraglutide can improve core MetS phenotypes (weight loss, glycemic control, improvement of insulin resistance, and dysregulation of glycolipid metabolism)	Level 1 evidence: Large-sample multicentre randomised controlled trials (RCTs) (Zhou et al., 2019; Davies et al., 2015; Mann et al., 2020; Mashayekhi et al., 2024)	No verification in models specific to stones	Liraglutide can significantly reduce body weight and glycated hemoglobin (HbA1c) in patients with type 2 diabetes/obesity, improve insulin sensitivity and glycolipid metabolism, and has confirmed clear cardiovascular and renal safety	All studies did not include nephrolithiasis as a prespecified endpoint, and it cannot be confirmed whether the improvement of metabolic phenotypes can be translated into clinical benefits for the prevention and treatment of nephrolithiasis
Liraglutide can attenuate apoptosis, programmed necrosis, and abnormal autophagy of RTECs, and maintain the structural integrity of renal tubules	Level 3 evidence: *In vitro* experiments in human renal tubular epithelial HK-2 cells, and *in vivo* experiments in animal models of diabetes/acute kidney injury (Wang et al., 2021; Zhao et al., 2015; Elkhoely, 2023)	No verification in models specific to stones	Liraglutide can attenuate high-glucose/metabolic stress-induced renal tubular epithelial cell injury and inhibit cell apoptosis, programmed necrosis, and abnormal autophagy through a GLP-1R-dependent pathway	All experiments are not specific to calcium oxalate stone models, and it has not been verified whether this renal tubular protective effect can reduce the adhesion and retention of calcium oxalate crystals
Liraglutide can regulate PGC-1α via the PKA/CREB and AMPK/SIRT1 pathways to improve the structural and functional homeostasis of renal tubular mitochondria	Level 3 evidence: *In vivo* experiments in animal models of acute kidney injury, obesity/diabetic kidney diseas (Wang et al., 2021; Elkhoely, 2023; Wang et al., 2018; Zhou et al., 2019)	No verification in models specific to stones	Liraglutide can upregulate PGC-1α expression, promote mitochondrial biogenesis, improve mitochondrial respiratory function, and alleviate mitochondrial structural damage and functional dysfunction	No verification in models specific to stones, and it has not been confirmed whether the improvement of mitochondrial homeostasis can reduce the susceptibility of RTECs to calcium oxalate crystal-induced injury
Liraglutide can inhibit the abnormal activation of the NLRP3 inflammasome, and attenuate inflammatory cascade reactions and pyroptosis	Level 3 evidence: *In vivo* animal experiments + *in vitro* cell experiments (Zhao et al., 2015; Yu X. et al., 2019; Zhu et al., 2018)	Partially relevant (only the pathological mechanism is related to lithogenesis)	Liraglutide can inhibit the activation of the NLRP3 inflammasome and the release of downstream IL-1β/IL-18 b y improving mitochondrial function and enhancing mitophagy, thereby blocking the inflammatory amplification cycle	No verification in models specific to stones; only extrapolated based on the theory that “mitochondrial injury-NLRP3 activation is the core pathophysiology of stones'; no direct experimental data of liraglutide in calcium oxalate crystal-induced inflammation models
Liraglutide can directly intervene in the nucleation, growth, adhesion, and deposition processes of calcium oxalate crystals	Level 4 (Indirect mechanistic evidence) (Xu et al., 2024)	No verification in models specific to stones	Mitochondrial ROS scavenging (Mito-Tempo) significantly reduces CaOx crystal deposition *in vivo*; however, no published direct experimental data confirms that liraglutide has this biological effect	No *in vitro* or *in vivo* specific to stones experimental data on liraglutide intervention; the direct effect of liraglutide on crystal adhesion and deposition remains to be verified
Liraglutide can reduce the risk of nephrolithiasis incidence and decrease postoperative stone recurrence	Level 4 evidence: Single-centre retrospective observational study, real-world database retrospective cohort study (Yeh et al., 2025; McCormick et al., 2024; Ganesan et al., 2025; Di Scipio et al., 2025)	No verification in models specific to stones	Existing clinical data can only confirm that GLP-1RAs have no superior stone-protective effect compared with SGLT2 inhibitors or no drug intervention; no clear positive/negative association between liraglutide use and the risk of nephrolithiasis incidence has been observed	No prospective RCT data with nephrolithiasis as a prespecified endpoint; it is impossible to distinguish the direct drug effect from the indirect effect of weight loss; the existing research sample size is limited, and confounding factors have not been fully adjusted

The upstream pharmacological cascade from GLP-1R activation to mitochondrial protection dependent on PGC-1α is robustly supported by preclinical data. In contrast, the downstream pathway linking improved mitochondrial function to reduced crystal adhesion and subsequent stone formation remains mechanistically plausible but is currently supported only by indirect inference at all steps beyond the mitochondrion.

### 5.5 Concluding remarks and implications

This review synthesizes evidence across endocrinology, nephrology and stone biology to propose a unifying working model: liraglutide, via coordinated activation of PKA/CREB and AMPK/SIRT1 signaling, upregulates PGC-1α in renal tubular epithelium, restores mitochondrial homeostasis, and thereby interrupts the “metabolism—inflammation—lithogenesis” cycle that drives nephrolithiasis associated with MetS.

The model remains a hypothesis. No trial has yet tested liraglutide against an endpoint specific to stones, and no experiment has tracked a calcium oxalate crystal from nucleation to clinical event under GLP-1 receptor activation. What makes the hypothesis worth testing is the strength of its foundation: the pharmacology of liraglutide is well-characterized, its renal safety is established (Davies et al., 2015; Mann et al., 2020), and the mitochondrial mechanisms it engages are biochemically mapped in relevant cell types (Section 3.1, Section 3.2, Section 3.3). Given that GLP-1RAs are increasingly prescribed to populations that overlap substantially with those at risk for nephrolithiasis, the question is no longer whether it should be asked, but how best to answer it.

Importantly, while the currently available clinical evidence supporting a direct anti-lithogenic effect of GLP-1RAs remains strictly limited and hypothesis-generating, the established safety profile of these agents offers immediate pragmatic utility. For patients with concurrent MetS and recurrent calcium oxalate stones, clinicians routinely face the choice of weight and glycemic control agents. The data reviewed herein demonstrate that GLP-1RAs do not worsen the lithogenic urinary profile (Feghali et al., 2024; Schaub et al., 2025), in contrast to malabsorptive bariatric surgery which markedly increases urinary oxalate excretion (Feghali et al., 2024). GLP-1RAs may also exert direct tubular protective effects via the mitochondrial pathways detailed in Section 3 and Section 4.

Collectively, prioritizing GLP-1RAs over other glucose-lowering or weight-loss medications in this specific patient population represents a reasonable, evidence-informed strategy that aligns metabolic management with stone prevention (Table 2). This recommendation remains provisional and must be balanced against considerations of cost, tolerability, and individual patient characteristics. Nonetheless, it represents a pragmatic approach that can be implemented immediately while higher-level evidence is generated.

Of the research directions outlined in Section 5.2, one emerges as the highest priority: validating the PGC-1α dependence of renal protection mediated by liraglutide using a nephron-specific knockout model. Crossing the NiPKO mouse (Svensson et al., 2016; Traykova-Brauch et al., 2008) with our validated composite model of MetS and stone disease would definitively address, in a single experimental series, whether the full mechanistic cascade from GLP-1R activation through PGC-1α and mitochondrial homeostasis to crystal deposition is causally required or merely correlative. A negative finding would redirect the field toward pathways independent of PGC-1α, while a positive result would provide the critical mechanistic proof of concept necessary to justify a dedicated stone-prevention trial.

Beyond this pivotal experiment, we propose two complementary research priorities. First, existing randomized controlled trial databases should be systematically interrogated for outcomes related to stones using standardized *post hoc* analytical approaches (Davies et al., 2015; Mann et al., 2020); even a retrospective signal would inform effect size estimates for future prospective trial design. Second, prospective biomarker studies correlating urinary mtDNA (Eirin et al., 2016), FGF21 and GDF15 with serial 24-h urine chemistry measurements would help bridge the gap between the mechanistic framework presented herein and clinically meaningful patient outcomes. Ultimately, whether this framework translates into a clinically viable strategy for stone prevention in metabolically compromised patients will depend on the rigorous execution of these proposed investigations.
